# Influence of Replacing Soybean Meal with *Nigella sativa* Seed Meal on Feed Intake, Digestibility, Growth Performance, Blood Metabolites, and Antioxidant Activity of Growing Lambs

**DOI:** 10.3390/ani14131878

**Published:** 2024-06-26

**Authors:** Ola G. A. Hassan, Noha A. Hassaan, Ahmed E. Kholif, Mireille Chahine, Gamal A. Mousa

**Affiliations:** 1 Animal Production Department, Faculty of Agriculture, Fayoum University, Fayoum 63514, Egypt; oga01@fayoum.edu.eg (O.G.A.H.); gam02@fayoum.edu.eg (G.A.M.); 2 Department of Animal Production, National Research Centre, 33 Bohouth St. Dokki, Giza 12622, Egypt; noha_a_h@hotmail.com; 3 Department of Animal Sciences, North Carolina Agricultural and Technical State University, Greensboro, NC 27411, USA; 4 Dairy Science Department, National Research Centre, 33 Bohouth St. Dokki, Giza 12622, Egypt; 5 Department of Animal, Veterinary and Food Sciences, University of Idaho, 315 Falls Ave., Twin Falls, ID 83301, USA

**Keywords:** amino acids, byproducts, protein feeds, economic evaluation, lambs, growth performance

## Abstract

**Simple Summary:**

Exploring alternative protein feeds (rich in phytonutrients) to replace a part of the conventional protein feeds in animal diets is an important issue in achieving sustainability and profitability for animal producers. *Nigella sativa* seed meal is one of the recommended alternatives to overcome the main problems facing the use of conventional protein feed: the high cost and fluctuation in price. *Nigella sativa* seed meal is rich in some bioactive compounds, in addition to considerable amounts of protein with low degradability and a good amino acids profile. In the present experiment, we aimed to partially or completely replace soybean meal with *N. sativa* seed meal. The results recommended the inclusion of *N. sativa* seed meal at about 12% of the total diet to replace about 75% of soybean meal for growing Ossimi lambs.

**Abstract:**

The present experiment aimed to evaluate the partial or complete substitution of soybean meal (SBM) with *Nigella sativa* seed meal (NSM) on chemical composition, in vitro ruminal fermentation, and the growth performance and economic efficiency of growing lambs. Thirty-two male Ossimi lambs weighing 41 ± 0.4 kg at 195 ± 5 d were divided randomly into four experimental groups of eight lambs each. Lambs were fed four diets containing 40% berseem clover and 60% concentrate feed mixture. Soybean meal was replaced with NSM at 0% (NSM0; control), 50% (NSM50), 75% (NSM75), or 100% (NSM100). The experiment lasted for 105 d, consisting of 15 d for adaptation and 90 days for measurements. Higher concentrations of crude protein (CP) and nonstructural carbohydrates were observed with SBM; however, NSM contained more fibers and gross energy. Moreover, SBM contained higher concentrations of individual amino acids and lower concentrations of polyphenols. The replacement did not affect in vitro gas production and decreased (*p* < 0.05) methane production and CP degradability. Treatments did not affect feed intake, nutrient digestibility, and diet’s nutritive value measured as starch value, total digestible nutrient, digestible energy, and apparent digestible crude protein. The NSM50 and NSM75 treatments increased (*p* < 0.001) total weight gain and daily gain compared to the control treatment, with lower feed conversion values associated with the NSM75 treatment. Treatments decreased cholesterol (*p* = 0.028) and high-density lipoprotein (*p* = 0.029) and increased antioxidant activity. Higher economic efficiencies were observed with the NSM75 followed by NSM50 and then NSM100 treatments. It is concluded that replacing 75% of SBM with NSM enhanced feed conversion and economic efficiency.

## 1. Introduction

Exploring alternative protein feeds such as *Nigella sativa* seed meal (NSM) to replace the high-cost available protein feeds such as soybean meal (SBM) is recommended to improve economic profit in livestock production. Soybean meal is the most common protein feed in livestock diets worldwide. Two main problems face livestock producers when SBM is used in diets: the high cost and fluctuation in price. Experiments evaluated many protein-rich alternatives, including sesame meal to replace 100% of SBM in diets of fatting Katahdin lambs [1], wheat germ meal to replace cottonseed meal at 100% in diets of Ossimi lambs [2], *Moringa oleifera* leaves meal to replace SBM at 100% in an in vitro experiment [3], sunflower meal to replace 50% of SBM in diets of Barki lambs [4], *N. sativa* meal to replace at 10% of the diet [5], and many other examples, with positive effects on animal performance. The replacement decreases the cost of feeding and production, resulting in more profitability for livestock producers.

The profile of amino acids and concentration of some bioactive compounds determine the nutritive value of a protein feed. One of the promising byproducts that may be included in the diet of animals is *N. sativa* meal. *Nigella sativa* is an herbal plant from the Ranunculaceae family. People use *N. sativa* mainly as a food additive or medicinal herb due to its contents of various active phytochemicals and vital nutrients [6,7,8]. The process of oil extraction from the seeds results in the production of a large biomass (e.g., meal), representing about 60 to 68% of the seeds [9].

*Nigella sativa* seed meal contains considerable amounts of protein (~7.5 to 33%), crude fibers (~6.5 to 20%), and carbohydrates (~35 to 68%) [7,9,10]. NSM protein has a low degradability and good amino acids profile [10]. *Nigella sativa* is not only a protein-rich feed but also contains various phytochemicals such as polyphenols and essential oils [8,11], which are beneficial for ruminant productivity [12]. The principal phenolic compounds in the seed are *p*-cymene and thymoquinone, which show antioxidant activities, including other beneficial attributes [10]. In addition, thymoquinone has immuno-modulatory properties and increases the activity of neutrophils as part of the natural body defense mechanism against invading infections [13].

Mahmoud and Bendary [14] completely substituted dietary protein by using NSM and sesame seed meal in the diets of growing lambs and calves. They did not observe significant differences between animals for final weight, total weight, and average daily gain of growing lambs. In other experiments, feeding NSM at 50 or 100 g/kg [5] or at 150 g daily [15] to Awassi ewes did not affect intakes of dry matter (DM) and crude protein (CP) but improved the digestibility of CP and neutral detergent fiber (NDF) and increased N retention as well as the final body weight and average daily gain of the lambs. The researchers also noted that the high level of inclusion increased daily milk production. In their meta-analyses, Sadarman et al. [12] concluded that increasing dietary NSM levels led to a linear increase in average daily gain and DM intake in lambs, as well as enhanced nitrogen digestion and immune responses.

Therefore, the present experiment aimed to evaluate the effects of replacing SBM with NSM at different levels in the diets of growing Ossimi lambs on the chemical composition of diets, in vitro ruminal fermentation, feed intake and digestion, blood parameters, growth performance, and economic efficiency. The hypothesis of this study was that the amino acid and polyphenol profiles of NSM would differently influence the digestion and utilization of feed compared to SBM, resulting in different feed conversion and growth performance of lambs.

## 2. Materials and Methods

### 2.1. Nigella sativa Seed Meal

*Nigella sativa* seed meal was collected from the Al-Jasmine factory for natural oils located in Fayoum Governorate, Egypt. *Nigella sativa* seed meal was prepared following the mechanical cold pressing extraction of oil from crushed ripe seeds, in accordance with the guidelines provided by Çakaloğlu et al. [16]. To ensure uniform mixing and to minimize animal selection, the *N. sativa* seed meal was sun-dried and ground before being added to the diets. Samples of SBM and NSM were individually ground prior to polyphenol and amino acid analysis at the Central Laboratory of the National Research Centre (Dokki, Giza, Egypt).

Polyphenol concentrations in SBM and NSM were measured using an Agilent 1260 series HPLC (Santa Clara, CA, USA). Before analysis, 1 g of each sample was soaked in 10 mL of 80% methanol. An Eclipse Plus C18 column (4.6 mm × 250 mm i.d., 5 μm) was used for the separation process. A full description of the process is given in Hassan et al. [2]. The mobile phase consisted of water (A) and 0.05% trifluoroacetic acid in acetonitrile (B) with a flow rate of 0.9 mL/min. The mobile phase was programmed with a linear gradient as follows: 0 min (82% A); 0–5 min (80% A); 5 to 8 min (60% A); 8 to 12 min (60% A); 12 to 15 min (82% A); 15 to 16 min (82% A); and 16 to 20 min (82% A). Detection was performed using a multi-wavelength detector set at 280 nm. Each sample solution was injected with a volume of 5 μL. The column temperature was maintained at 40 °C, and a standard from Sigma-Aldrich GmbH, (Steinheim, Germany) was utilized.

Amino acid profiles in SBM and NSM were analyzed using an Agilent 1260 series HPLC (Santa Clara, CA, USA) following the method described by Jajic et al. [17]. The separation was conducted with an Eclipse Plus C18 column (4.6 mm × 250 mm i.d., 5 μm). A full description of the process is given in Hassan et al. [2]. Specifically, 0.1 g of each sample was mixed with 2.5 mL of H_2_O and 2.5 mL of 6 M HCl and then heated at 100 °C for 24 h before filtration. Finally, 1 mL of the filtrate was dried and resuspended in 0.1 M HCl before being injected into the HPLC system. The separation was carried out using an Eclipse Plus C18 column (4.6 mm × 250 mm i.d., 5 μm). The mobile phase consisted of sodium phosphate dibasic and sodium borate buffer at pH 8.2 (A) and ACN:MeOH:H_2_O 45:45:10 (B) at a flow rate of 1.5 mL/min. The Diode Array Detector (DAD) was monitored at 338 nm and Bandwidth 10 nm. The fluorescence detector was adjusted as follows: from 0 to 27 min, at 340/450 nm (Excitation/Emission), and from 27 to 35 min, at 266/306 (Excitation/Emission). Amino acid standards obtained from Sigma (Product #A6282, Saint Louis, MO, USA) were used.

### 2.2. In Vitro Fermentation

Using a stomach tube, rumen liquor (about 500 mL) was obtained from three adult Barki sheep (50 ± 1.9 kg of body weight) fed a fixed amount of concentrate (500 g) and *ad libitum* berseem (*Trifolium alexandrinum*) hay daily. The rumen contents were collected before morning feeding and kept in pre-warmed thermo containers at 39 °C under anaerobic conditions. The first 50 mL of the rumen fluid samples were discarded to avoid saliva contamination. The rumen fluid was mixed for 10 s, squeezed through four layers of cheesecloth, and maintained in a water bath at 39 °C under continuous CO_2_ flushing until inoculation [18]. Three incubation runs were performed in three different weeks. Rumen contents obtained from the three sheep were combined for each run.

Four diets containing 40% berseem clover and 60% concentrate feed mixture were formulated. The control diet (NSM0) did not include any NSM, whereas NSM replaced SBM in the other diets at 50% (NSM50), 75% (NSM75), or 100% (NSM100). The same diets were evaluated in the in vivo experiment and fed to lambs. The ingredients are listed in Table 1, while the composition of the diets is detailed in Table 2. The in vitro total gas production assay was conducted as described by Theodorou et al. [18] using a digital pressure manometer (Extech instruments, Waltham, MA, USA). Ground substrate samples (500 mg of DM) of all evaluated treatments were incubated in 120 mL serum bottles (3 bottles per treatment). Bottles without substrate and containing only buffered rumen liquor were considered blanks. Upon completion of the incubation at 48 h, 5 mL of headspace gas was extracted from each bottle and introduced into a Gas-Pro detector (Gas Analyzer CROWCON Model Tetra3, Abingdon, UK) to quantify the CH_4_ concentration. Both the control and experimental diets underwent testing in 3 bottles (analytical replicates) across 3 consecutive weeks of incubation runs. Additionally, 3 bottles containing inoculum and buffer without feed were served as blanks).

At the end of incubation at 48 h, fermentation was halted by placing the bottles in ice. For each treatment, 3 bottles were utilized to measure the pH and short-chain fatty acids (SCFAs) via steam distillation and titration [19]. Meanwhile, the remaining 3 bottles were filtered in pre-weighed crucibles and washed with hot water followed by acetone. Subsequently, the residual DM and ash were measured to determine true organic matter (OM), CP, NDF, and acid detergent fiber (ADF) degradability (*d*OM, *d*CP, *d*NDF, and *d*ADF, respectively).

### 2.3. Animal Management and Experimental Design

This experiment was performed at the Faculty of Agriculture experimental farm. All chemical analyses were performed at the laboratories of the Animal Production Department, Fayoum University, and the National Research Centre. The farm experiences a hot and dry climate, with infrequent winter rain and average daily temperatures ranging from 9 to 23 °C. The experimental protocol and animal procedures were approved by the Fayoum University Institutional Animal Care and Use Committee (FU-IACUC) under proposal code number: AEC2311.

Thirty-two healthy and clinically free of internal and external parasites male Ossimi lambs (weighing 41 ± 0.4 kg) at 195 ± 5 d were divided randomly into four experimental groups of eight lambs each based on their live body weight at allocation. Prior to commencing the experiment ‘the adaptation period’, lambs were vaccinated against clostridium using Covaxin 10 (Merck & Co., Inc., Rahway, NJ, USA) administered subcutaneously at 1 cm, with repeat dose after 21 days, followed by subsequent vaccinations every 6 months. Additionally, lambs were treated with Ivomec Super (Boehringer Ingelheim, Bracknell, UK) via subcutaneous injection at 2 cm. Lambs were kept outdoors with shelter during the day and housed in semi-open barns at night under the same environmental and management conditions. Light bulbs were used to provide 12 h of light per day for all lambs. Each lamb in the trial was kept in an area of 2 × 1.5 m^2^.

Lambs were fed a diet comprising 40% berseem clover and 60% concentrate feed mixture as the control diet (NSM0). Soybean meal was replaced with NSM at three levels, 50% (NSM50), 75% (NSM75), or 100% (NSM100), corresponding to inclusion levels of 7.8%, 11.7%, and 15.6%, respectively. The rations were formulated according to the guidelines provided by the NRC [20]. The experiment spanned 105 d, with an initial 15 d adaptation period followed by 90 days for measurements. Lambs received the concentrate mixture at 08:00 h, while berseem clover was offered at 16:00 h, with continuous access to fresh water. Samples of concentrates mixture and berseem clover were collected daily, composited weekly, dried at 60 °C in a forced-air oven for 24 h, and stored for subsequent chemical analysis. Feed intake was recorded daily by weighing the offered diets and refusals from the previous day. Additionally, lambs were individually weighed biweekly before the morning feeding.

**Table 2:** Chemical composition (g/kg DM) of feedstuff and total mixed rations fed to the Ossimi lambs.

	Diet ^1^				Ingredients				
	NSM0	NSM50	NSM75	NSM100	Berseem Clover	NSM	Soybean Meal	Yellow Corn	Wheat Bran
OM	869	867	867	866	829	906	934	913	896
CP	168	166	166	165	152	351	415	107	139
EE	35	41	43	46	43	89	18	36	32
NFCs	442	427	420	412	264	196	383	693	431
NDF	223	233	238	243	370	270	118	77	294
ADF	136	145	150	154	149	196	71	17	110
Hemicellulose	87	88	88	89	221	74	47	60	184
GE (Mcal/kg DM) ^2^	4.11	4.13	4.13	4.16	4.02	4.78	4.57	4.18	4.16

^1^ The control diet based on (per kg DM): 600 g of concentrates feed mixture and 400 g berseem clover (*Trifolium alexandrinum*) (NSM0 diet). *Nigella sativa* seed meal (NSM) was included at 7.8%, 11.7%, or 15.6% to replace soybean meal at 50% (NSM50 diet), 75% (NSM75 diet), or 100% (NSM100 diet), respectively. ADF = acid detergent fiber, CP = crude protein, EE = ether extract, GE = gross energy, NDF = neutral detergent fiber, NFCs = non-fibrous carbohydrates, OM = organic matter. ^2^ Calculated according to MAFF [21].

### 2.4. Digestibility Trial

At the end of the experiment, a digestibility trial was conducted where lambs were individually maintained in metabolic cages (70 cm width × 150 cm length ×120 cm height) for 7 consecutive days. Daily feces excreted from each lamb were weighed, and 10% of fresh feces was collected and dried at 60 °C for 24 h to determine the DM content of the feces. Composite samples from the daily dried feces of each lamb were mixed, ground, and stored in a refrigerator for subsequent chemical analysis.

Feed and feces samples were analyzed for dry matter (method 930.15), ash (method 942.05), ether extract (method 920.39), and N content (Kjeldahl method 955.04) according to the official methods of the AOAC [22]. Acid detergent fiber (ADF) and neutral detergent fiber (NDF) of feed were determined according to Van Soest et al. [23]. Non-fibrous carbohydrates (NFCs; 1000 − NDF − CP − EE − ash), hemicelluloses = (NDF − ADF), and OM (100 − ash) were calculated. Moreover, acid-insoluble ash was used as an internal indigestibility marker, and coefficients of digestion were determined according to Van Keulen and Young [24].

Starch value (SV), total digestible nutrient (TDN), digestible energy (DE), and apparent digestible crude protein (DCP) were calculated according to the equations of NRC [25] as: apparent SV = (apparent DCP (g/kg DM) × 0.94) + (digestible crude fiber (g/kg DM) × 1) + (digestible nitrogen free extract (g/kg DM) × 1) + (digestible EE (g/kg DM) × 2.09); true SV (g/kg DM) = apparent SV − digestive effort for fiber degradation by animal; TDN (g/kg DM) = apparent DCP (g/kg DM) + digestible crude fiber (g/kg DM) + digestible nitrogen free extract (g/kg DM) + digestible EE (g/kg DM) × 2.25; apparent DCP (g/kg DM) = CP (g/kg DM) × CP digestibility (g/kg DM); DE (Mcal/kg DM) = 0.04409 × TDN (%); and metabolizable energy (ME; Mcal/kg DM) = DE × 0.82.

### 2.5. Blood Sampling and Analysis

Blood samples were collected at the end of the experimental period, all before the morning feeding. Approximately 8 mL of fresh blood was withdrawn from the jugular vein of each lamb using 10 mL syringes. After removing the needle cap, each blood sample was divided into two aliquots in 5 mL sterile plastic disposable screw-capped tissue culture tubes by gently pressuring the syringe plunger. One tube was treated with sodium fluoride and potassium oxalate for glucose analysis, while the other was left without additives for serum separation. The blood samples were then centrifuged for 15 min at 3500 rpm to separate serum. The clear serum samples were transferred into 2 mL Eppendorf tubs and stored at −20 °C until analysis. Specific kits from Linear Chemicals S.L. (Montgat, Barcelona, Spain) were used to analyze the samples according to the manufacturer’s instructions.

Blood serum samples were spectrophotometrically (T80 UV/VIS PG instrument Ltd., Lutterworth, UK) analyzed for albumin (g/dL), globulin (mg/dL), total protein (g/dL), triglycerides (mg/dL), high-density lipoprotein (HDL, mg/dL), total cholesterol (mg/dL), creatinine (mg/dL), urea-N (mg/dL), aspartate aminotransferase (AST, IU/L), and alanine aminotransferase (ALT, IU/L). Globulin samples were calculated by subtracting the obtained albumin value from their corresponding total protein value. The serum antioxidant activity capacity was assessed using 1, 1-diphenyl-2-picrylhydrazyl (DPPH) reduction assay provided by Himedia Laboratories Pvt., Ltd., Maharashtra, India [26]. The assay was conducted by mixing 20 µL of serum with 10 mM buffer of sodium phosphate (pH 7.4) to a total volume of 400 µL, then adding this mixture to 400 µL of 0.1 mM methanol solution of DPPH. After incubation at 21 °C for 30 min, the absorbance of samples (Abs sample) at 520 nm was measured. This absorbance was compared to that of a control sample containing only phosphate buffer and DPPH solution (Abs control), as well as a blank sample containing only serum and phosphate buffer (Abs blank). The antioxidant activity (AA%) was calculated using the following equation: AA%= 100 − {(Abs sample − Abs blank × 100)/Abs control} [27].

### 2.6. Economic Evaluation

The economic return of the evaluated values was calculated based on the price of feed ingredients during the study, assuming that the price of 1 kg live body weight gain of lambs was USD 4.4 and the cost of 1 ton of berseem clover (15% DM) in all the tested diets was USD 11, while the costs of one ton of concentrate feed mixture were USD 315, USD 303, USD 295, and USD 284 (on DM basis) for the diets containing NSM at 0%, 50%, 75%, and 100%, respectively.

### 2.7. Statistical Analyses

The lambs were randomly assigned to four experimental groups in a completely randomized design. Prior to statistical analysis, data were assessed for normal distribution, confirming that all measurements exhibited normal distributions. Weekly generated data were analyzed using the PROC MIXED procedure of SAS, treating period as a repeated measure and individual lambs as the experimental unit. The model used for the analysis was Y_ijkl_ = μ + D_i_ + A_j_(D_i_) + P_k_ + (D × P)_ik_ + e_ijkl_, where Y_ijkl_ = observation of the jth lamb in the kth sampling time given ith treatment, D_i_ = diet effect, A_j_(D_i_) = lamb within treatments, P_k_ = sampling period effect, (D × P)_ik_ = interaction between the treatments and sampling week, and e_ijkl_ = experimental error. Data collected once (i.e., blood and digestibility) were analyzed using the model Y_ijk_ = μ + D_i_ + A_j_(D_i_) + e_ijk_, where Y_ijk_ = observation of the jth lamb given ith treatment, D_i_ = treatments effect, L_j_(D_i_) = lamb within treatments, and e_ijk_ = experimental error. Polynomial contrasts (linear and quadratic) were applied to assess responses to increasing levels of NSM and to compare the control vs. the average of NSM treatments. Significance was declared at a level of *p <* 0.05.

## 3. Results

### 3.1. Chemical Composition

The chemical composition differed between NSM and SBM (Table 2). Soybean meal contained more CP (41.5 vs. 35.1%) and NFCs (38.3 vs. 19.6%) than NSM, which had more EE (8.9% vs. 1.8%), NDF (27.0 vs. 11.8%), ADF (19.6 vs. 7.1%), hemicellulose (7.4 vs. 4.7%) and GE (4.78 vs. 4.57 Mcal/kg DM).

Soybean meal contained a completely different amino acid profile to NSM. Soybean meal contained higher (*p* < 0.05) total (32.33 vs. 23.81%), individual amino acids (except arginine, glutamic acid, and tyrosine), and total essential (11.94 vs. 6.95%) (*p* < 0.001) and nonessential (20.39 vs. 16.86%) (*p* = 0.009) amino acids (Table 3).

*Nigella sativa* seed meal contained higher concentrations of total polyphenols (*p* = 0.003), including gallic acid (*p* = 0.005), chlorogenic acid (*p* < 0.001), methyl gallate (*p* < 0.001), coffeic acid (*p* = 0.022), rutin (*p* = 0.009), ferulic acid (*p* = 0.009), rosmarinic acid (*p* < 0.001), vanillin (*p* = 0.003), quercetin(*p* < 0.001), and cinnamic acid (*p* < 0.001); however, SBM contained more syringic acid (*p* = 0.003), and naringenin (*p* = 0.015) (Table 4).

### 3.2. In Vitro Ruminal Fermentation

Replacing SBM did not affect gas and CO_2_ production, pH value, or SCFAs production (Table 5). However, NSM-containing diets linearly decreased (*p* = 0.030) CH_4_ production. The NSM75 and NSM100 treatments linearly and quadratically decreased *d*CP (*p* = 0.046) without affecting other nutrient degradabilities.

### 3.3. Feed Intake, Growth Performance, and Feed Conversion

Treatments did not affect intakes of DM, SV, TDN, or DE; however, the NSM100 quadratically (*p* = 0.009) decreased the intake of DCP (Table 6).

With no differences between the initial of lambs, the NSM50 and NSM75 treatments linearly increased final body weights (*p* = 0.034), total weight gain (*p* = 0.001), and daily gain (*p* = 0.001) compared to the control and NSM100 treatments (Table 6).

The least feed conversion values (quadratic effects) calculated as kg DM intake/kg gain (*p* = 0.044), kg TDN intake/kg gain (*p* = 0.022), kg SV intake/kg gain (*p* = 0.045), g DCP intake/g gain (*p* = 0.035), Mcal DE intake/kg gain (*p* = 0.039), and Mcal ME intake/kg gain (*p* = 0.049) were observed with the NSM75 treatment compared to the control and other treatments (Table 6).

### 3.4. Diets Digestibility and Nutritive Value

Replacing SBM with NSM did not affect nutrient digestibility or diet nutritive value expressed as true SV, TDN, DCP, DE, or ME (Table 7).

### 3.5. Blood Measurements

Treatments did not affect the concentrations of blood total protein, albumin, globulin, urea-N, creatinine, glucose, alanine transaminase, aspartate transaminase, or triglycerides (Table 8). However, treatments linearly decreased cholesterol (*p* = 0.028) and high-density lipoprotein (*p* = 0.029), and linearly increased antioxidant activity (*p* = 0.030). The contrasts between the control and the average for the three NSM diets (NSM0 vs. others) for cholesterol, HDL, and antioxidant activity were significant (*p* < 0.05).

### 3.6. Economic Evaluation

Replacing SBM with NSM gradually decreased (linear and quadratic effects, *p* < 0.001) the cost of kg DM of the diet and the cost of feed consumed (Table 9). In addition, the replacement of SBM with NSM linearly increased (*p* < 0.001) the net revenue and relative percentage of net revenue, with the highest value observed with the NSM75 treatment followed by the NSM50 treatment and then, the NSM100 treatment (*p* < 0.001).

## 4. Discussion

### 4.1. Chemical Composition

Soybean meal contained higher concentrations of CP and NFCs compared to NSM, indicating higher nutritive value of SBM compared to NSM. However, this effect was not pronounced as will be discussed later. Other factors, such as the profile and concentrations of amino acids, protein degradability, and polyphenols may overcome such low protein content.

The amino acid profile completely differed between SBM and NSM, with higher concentrations in SBM. In the present experiment, glutamic acid followed by aspartic acid, leucine, arginine, serine, and glycine were the principal amino acids in NSM; however, Bewley et al. [28] stated that arginine, aspartic acid, and glutamic acid are the main amino acids in the seed with small contents of cysteine and methionine. Differences between the present results and those reported in the literature may be attributed to various factors, including cultivars, environmental conditions, interactions between cultivars and environment agricultural practices during plant growth (e.g., fertilization), and harvest and post-harvest treatments of soybean plants [29]. Soybean meal contained higher concentrations of total amino acids by 26.4%, total essential by 41.8%, and nonessential amino acids by 17.3% compared to NSM, indicating the superior amino acid profile of SBM. However, as observed in the in vitro experiment, NSM had lower *d*CP compared to SBM. The balance between concentrations of amino acids and *d*CP determines the nutritive value of NSM and SBM as protein feeds. Absorbed (not ingested) amounts of amino acids serve as building blocks for proteins in the muscles and are substrates for the synthesis of low-molecular-weight substances [30].

*Nigella sativa* seed meal contained higher concentrations of polyphenols than SBM, which may justify, at least in part, the differences in results between NSM- and SBM-containing diets and also the high and low inclusion levels of NSM. Polyphenols at appropriate levels cause positive effects on feed digestion and ruminal fermentation compared to the high levels of inclusion, which impair ruminal fermentation [31,32]. Moreover, the presence of higher concentrations of polyphenols in NSM may partially explain its low ruminal *d*CP as will be discussed later.

### 4.2. In Vitro Ruminal Fermentation

Replacing SBM had a weak effect on gas and CO_2_ production; however, it decreased CH_4_ production, causing qualitative changes in the produced gases. The lowered CH_4_ production with NSM treatments may be related to its contents of polyphenols with antimicrobial effects on ruminal protozoa and CH_4_-producing bacteria [31]. Feeding NSM at 1.2% to Barbarine lambs [11] and replacing cottonseed meal with NSF at 33.3 and 66.7% in diets of Farafra lambs [33] decreased the number of ruminal protozoa. As previously mentioned, the seed of *N. sativa* contains thymoquinone and thymohydroquinone, which possess antimicrobial properties against many ruminal microbes [10].

Minimal effects were observed on pH value and SCFAs production when NSM replaced SBM. El-Tanany et al. [4] observed unchanged ruminal pH and SCFAs production when one-half of SBM was replaced with NSM in the diets of Barki lambs. Regarding nutrient degradability, both NSM75 and NSM100 decreased ruminal *d*CP without affecting other nutrient degradability, which may be related to the presence of phenolic compounds in NSM [10,31,32]. Secondary metabolites in NSM may cause changes in the composition of the ruminal microbes, resulting in a reduction in the number and activity of CH_4_-producing microbes [34]. Such effects are desirable since they will increase the amounts of protein that bypass the rumen and are absorbed in the small intestine. Jayanegara et al. [35] claimed a negative correlation between total phenol and CH_4_ production. Additionally, the high content of fat and fatty acids in NSM compared to SBM may be considered as another reason for decreasing CH_4_ production due to their ability to reduce the activity of rumen CH_4_-producing bacteria and change the process of biohydrogenation [36]. The effect of NSM levels on CH_4_ production was not pronounced, indicating that the used levels of NSM in the evaluated diets were enough to suppress CH_4_ production.

High levels of replacing SBM with NSM (i.e., NSM75 and NSM100 treatments) decreased nutrient degradability, revealing that the presence of polyphenols in NSM at high concentrations decreases ruminal degradation of the protein and increases protein bypass to the intestine to be absorbed as a true protein.

### 4.3. Feed Intake, Growth Performance, and Feed Conversion

Even though feeding NSM showed weak effects on intake and digestibility, the NSM50 and NSM75 treatments increased final weight gain, total weight gain, and daily gain. This indicates that the changes in the daily gain are not due to protein contents in the diet or feed intake but mainly due to the bioactive components in NSM and protein degradability [12,37]. In their experiment, Sadarman et al. [12] reported a positive relationship between feeding NSM to lambs and their daily gain, attributing this to the phytochemical composition and protein content of the seeds. They suggested several mechanisms for the positive effects of NSM as growth promoters including their roles in modulating rumen fermentation and microbiota, enhancing enzyme secretion, improving nutrient digestion and absorption, and boosting immunity. Singh et al. [10] stated that the antioxidant activity of the polyphenols in NSM seed enhances daily gain at suitable levels. Low protein degradability with NSM-containing diets decreases ruminal protein degradation and increases the amounts of true protein that reach the small intestine and is absorbed to serve as building blocks for muscle proteins [30]. Moreover, the profile of fatty acids in NSM as an energy source may be another reason [12,38]; however, we did not study the latter. *Nigella sativa* meal is rich in linolenic, oleic acid, and linoleic acid, all of which are necessary for body growth [39].

The enhanced daily gain and unchanged feed intake in the NSM75 treatment were reflected as an enhanced feed conversion compared to other treatments. Obeidat et al. [5] observed unchanged intakes of DM, CP, NDF, ADF, and N, and increased final body weight and daily gain of ewes fed NSM at 50 and 100 g daily. Moreover, El-Ghousein [40], Obeidat [15], and Retnani et al. [41] observed increased daily gain in lambs fed a diet supplemented with NSM at 10 g, 150 g, and 20% (equals to 172.9 g), respectively.

### 4.4. Diets Digestibility and Nutritive Value

Treatments did not affect nutrient digestibility. Consistent with the present results, El-Tanany et al. [4] and Cherif et al. [11] observed unchanged apparent digestibility of DM, OM, CP, and NDF when feeding *N. sativa* seeds to lambs. Many other experiments showed that replacing protein feed in the diet with NSM or the administration of NSM directly as a feed additive improved nutrient digestibility or at least CP digestibility [5,33,41]. It was expected that the presence of polyphenols at higher concentrations in NSM would increase the digestibility of CP and fibers due to probably enhancing rumen microorganisms and their digestion capabilities with the presence of polyphenols [12,31,32]. The minimal effects of treatments on nutrient digestibility were reflected as unaffected diet nutritive value. Similar results on TDN and DCP were observed by El-Tanany et al. [4].

### 4.5. Blood Measurements

The measured blood parameters in the present experiment were within the established reference ranges for healthy animals [42]. The weak effects of treatments on blood total protein, albumin, globulin, urea-N, and creatinine indicate normal kidney and liver functions and unaffected metabolic status of lambs fed NSM since they are indicators for the amino acid metabolic status of ruminants [43]. No significant differences were reported by El-Hawy et al. [44] for total protein, albumin, and globulins in Bakri ewes fed on NSM as a protein feed. Obeidat et al. [5] did not observe any changes in blood parameters with feeding ewes 50 and 100 g NSM daily. The weak effects of treatments on blood alanine transaminase and aspartate transaminase, and values within the reference range for healthy lambs [45] are other indicators of unaffected liver functions with feeding NSM.

Replacing SBM with NSM decreased cholesterol and high-density lipoprotein, which may be related to the polyphenols present in NSM, which can act as an excellent superoxide anion scavenger for free radicals [46]. Additionally, polyphenols act on the cellular antioxidant signaling pathway, activating related transcription factors and regulating the expression of downstream genes [34]. The concentration of unsaturated fatty acids in NSM may be another reason for decreasing cholesterol and high-density lipoprotein [47]. Abdullah and Farghaly [33] observed lowered concentrations of cholesterol and liver enzymes when feeding lambs on diets containing NSM.

Replacing SBM with NSM increased antioxidant activity. This may be related to the presence of secondary metabolites and polyphenols in the NSM, which function as antioxidants and stimulate the oxidative stress response as they scavenge free radicals and ameliorate the oxidative damage, they cause to cells [46]. Moreover, polyphenols act on the cellular antioxidant signaling pathway, activating related transcription factors and regulating the expression of downstream genes [34].

### 4.6. Economic Evaluation

Feed costs represent the most significant recurring expense in livestock production, especially with protein feeds being the most expensive. By minimizing these feeding costs, producers can expect a positive impact on their net revenue [48,49].

Replacing SBM with NSM decreased the cost of feeding lambs and increased the net revenue and relative percentage of net revenue with the highest efficiency for the NSM75 treatment. In the present study, the diets containing NSM (i.e., NSM50, NSM75, and NSM100) decreased feed cost by 13%, 17%, and 22%, respectively, compared to the control diet. Additionally, these NSM diets increased the net revenue by 42.3%, 64.3%, and 26.6%, respectively, in comparison to the control diet. The improvement in economic efficiency with feeding NSM is mainly due to the low cost of NSM compared to SBM and the increased total weight gain caused by the addition of NSM. Using NSM as a protein feed supports farmer income by producing heavier animals with low-cost feeding. Obeidat et al. [5] observed lowered feeding costs of 6 and 11.3%, respectively, when ewes were fed on 50 and 100 g NSM daily. In another experiment, Obeidat [15] reported that the cost/kg of weight gain was decreased by 31% when Awassi lambs were fed 150 g NSM as a partial replacement of barley grain and SBM.

## 5. Conclusions

Soybean meal contains more protein than *N. sativa* meal, which has lower ruminal degradability with different profiles of amino acids. Soybean meal can be partially replaced with *N. sativa* meal as a protein feed in the diet of growing lambs. Replacing 75% of soybean meal increased feed efficiency, growth performance, and economic efficiency compared to other replacement levels. Hence, using NSM to replace SBM as an alternative protein feed for feeding Ossimi lambs is economically beneficial. More long-term studies on animals are needed to verify its effect and explore the specific mechanism of action on the performance of animals at different growth stages. Moreover, the impact of replacing SBM with NSM on consumers and future perspectives should be considered in further experiments.

## Figures and Tables

**Table 1:** Ingredients (g/kg DM) used to formulate the experimental diets fed to the Ossimi lambs.

	Diet ^1^			
	NSM0	NSM50	NSM75	NSM100
Berseem clover	400	400	400	400
Yellow corn	342	342	342	342
Wheat bran	114	102	96	90
Soybean meal	132	66	33	0
NSM	0	78	117	156
Limestone	6	6	6	6
Premix	3	3	3	3
NaCl	3	3	3	3

^1^ The control diet based on (per kg DM): 600 g of concentrates feed mixture and 400 g berseem clover (*Trifolium alexandrinum*) (NSM0 diet). *Nigella sativa* seed meal (NSM) was included at 7.8%, 11.7%, or 15.6% to replace soybean meal at 50% (NSM50 diet), 75% (NSM75 diet), or 100% (NSM100 diet), respectively.

**Table 3:** Amino acid (AA) profile of soybean meal (SBM) and *Nigella sativa* seed meal (NSM) (g/kg DM) (n = 3 samples).

	SBM	NSM	SBM vs. NSM (Change % ^1^)	SEM	*p*-Value
Essential AA					
Histidine	9.4	8.59	8.6	0.35	0.022
Threonine	13.6	10.2	24.8	0.70	0.016
Valine	15.5	9.61	38.0	0.83	0.010
Methionine	5.40	4.32	20.0	0.14	0.029
Phenylalanine	17.2	9.19	46.6	1.66	0.010
Isoleucine	16.1	8.46	47.5	1.62	<0.001
Leucine	30.5	14.2	53.6	3.12	<0.001
Lysine	11.7	4.92	58.1	2.42	<0.001
Nonessential AA					
Aspartic acid	37.9	24.9	34.4	2.57	0.001
Glutamic acid	69.2	69.1	0.1	0.36	0.937
Serine	16.5	12.1	26.5	1.42	0.005
Glycine	15.4	15.3	0.6	0.17	0.919
Arginine	24.0	23.5	2.1	1.39	0.810
Alanine	14.8	10.7	27.9	1.64	0.019
Tyrosine	10.0	10.6	−5.9	0.22	0.308
Cystine	1.64	ND	100	0.18	<0.001
Proline	14.5	2.43	83.4	2.12	<0.001
Σ Essential AA	119.4	69.5	41.8	6.72	<0.001
Σ Nonessential AA	203.9	168.6	17.3	14.37	0.006
Total AA	323.3	238.1	26.4	18.02	0.004

ND = not detected. ^1^ Change % refers to the difference in concentration between SBM and NSM.

**Table 4:** Concentration ^1^ (µg/g) of polyphenols of soybean meal (SBM) and *Nigella sativa* seed meal (NSM) identified by HPLC analysis (n = 3 samples).

Compound ^2^	SBM	NSM	SBM vs. NSM (Change % ^3^)	SEM	*p*-Value
Gallic acid	79.1	136.0	−72	5.30	0.005
Chlorogenic acid	36.4	361.6	−893	6.96	<0.001
Methyl gallate	18.0	295.7	−1543	4.27	<0.001
Coffeic acid	63.2	70.6	−11.7	1.68	0.022
Syringic acid	78.0	3.4	95.6	3.63	0.003
Rutin	48.3	801.0	−1558	26.99	0.009
Vanillin	78	92.2	−18.2	4.40	0.003
Ferulic acid	2.0	2.2	−10.0	0.10	0.009
Naringenin	28.3	12.5	55.8	0.72	0.015
Rosmarinic acid	ND	39.4	−100	2.58	<0.001
Quercetin	ND	12.6	−100	1.50	<0.001
Cinnamic acid	ND	1.8	−100	0.09	<0.001
Σ Polyphenols	431.3	1829	−324	40.21	0.003

ND = not detected. ^1^ Concentration based on the total areas of the identified peaks. ^2^ Identification based on authentic standards, the National Institute of Standards and Technology (NIST) library spectra, and the literature. ^3^ Change % refers to the difference in concentration between SBM and NSM.

**Table 5:** In vitro evaluation of diets with different levels of *Nigella sativa* seed meal replacing soybean meal as a protein source.

	Diet ^1^				SEM	*p*-Value			
	NSM0	NSM50	NSM75	NSM100		Diet	NSM0 vs. Others	Linear	Quadratic
Gas production (mL/g DM)	192.0	184.6	189.2	188.9	2.47	0.329	0.225	0.246	0.427
Methane (mL/g DM)	50.4 ^a^	47.3 ^b^	47.1 ^b^	45.7 ^b^	0.83	0.035	0.039	0.030	0.316
Carbone dioxide (mL/g DM)	130.6	135.4	130.2	131.2	6.38	0.846	0.691	0.451	0.866
pH value	5.93	5.90	5.93	5.97	0.029	0.487	1.000	0.620	1.000
SCFAs (mmol/g DM)	25.5	26.2	27.2	26.0	1.48	0.312	0.339	0.471	0.215
*d*OM (%)	53.4	50.0	47.7	44.9	2.93	0.038	0.041	0.052	0.122
*d*CP (%)	51.3 ^a^	48.9 ^ab^	45.2 ^b^	44.3 ^b^	1.88	0.046	0.041	0.033	0.013
*d*NDF (%)	49.8	47.7	45.4	44.6	2.92	0.231	0.212	0.232	0.329
*d*ADF (%)	45.2	40.3	40.4	39.2	21.58	0.340	0.342	0.344	0.332

^a,b^ Means in the same row with different superscripts differ (*p* < 0.05). *p*-value is the observed significance level of the F-test for treatment; SEM = standard error of the mean. ^1^ The control diet based on (per kg DM): 600 g of concentrates feed mixture and 400 g berseem clover (*Trifolium alexandrinum*) (NSM0 diet). *Nigella sativa* seed meal was included at 7.8%, 11.7%, or 15.6% to replace soybean meal at 50% (NSM50 diet), 75% (NSM75 diet), or 100% (NSM100 diet), respectively. *d*OM is organic matter degradability (%), *d*CP is crude protein degradability (%), *d*NDF is neutral detergent fiber degradability (%), *d*ADF is acid detergent fiber degradability (%), SCFAs is short-chain fatty acids (mmol/g DM).

**Table 6:** Feed intake, growth performance, and feed conversion of Ossimi lambs fed diets with different levels of *Nigella sativa* seed meal replacing soybean meal as a protein source.

	Diet ^1^				SEM	*p*-Value			
	NSM0	NSM50	NSM75	NSM100		Diet	NSM0 vs. Others	Linear	Quadratic
Feed intake									
DM (kg/lamb/d)	1.64	1.62	1.65	1.61	0.242	0.323	0.339	0.317	0.438
SV (kg/lamb/d)	1.04	1.05	1.09	1.00	0.100	0.151	0.250	0.062	0.244
TDN (kg/lamb/d)	1.10	1.11	1.15	1.06	0.015	0.236	0.290	0.096	0.390
DCP (g/lamb/d)	211.6 ^a^	205.3 ^a^	209.7 ^a^	192.5 ^b^	2.79	0.023	0.019	0.276	0.009
DE (Mcal/lamb/d)	4.86	4.90	5.06	4.65	0.053	0.167	0.272	0.068	0.269
Growth performance									
Initial weight (kg)	41.0	41.1	41.4	41.3	1.20	0.995	0.837	0.855	0.979
Final weight (kg)	60.0 ^b^	61.8 ^a^	63.1 ^a^	59.8 ^b^	0.86	0.034	0.136	0.007	0.354
Total weight gain (kg/lamb)	19.0 ^b^	20.6 ^a^	21.7 ^a^	18.4 ^b^	0.48	0.001	0.034	<0.001	0.091
Daily gain (g/lamb)	211.5 ^b^	230.0 ^a^	241.1 ^a^	205.6 ^b^	5.34	0.001	0.034	<0.001	0.091
Feed conversion									
kg DM intake/kg gain	7.75 ^a^	7.04 ^a^	6.84 ^b^	7.83 ^a^	0.411	0.032	0.049	0.189	0.044
kg TDN intake/kg gain	5.20 ^a^	4.83 ^a^	4.77 ^b^	5.16 ^a^	0.225	0.026	0.041	0.135	0.022
kg SV intake/kg gain	4.92 ^a^	4.57 ^a^	4.52 ^b^	4.86 ^a^	0.212	0.026	0.045	0.136	0.045
g DCP intake/g gain	1.00 ^a^	0.89 ^a^	0.84 ^b^	0.94 ^a^	0.050	0.036	0.045	0.301	0.035
Mcal DE intake/kg gain	23.0 ^a^	21.3 ^a^	20.8 ^b^	22.6 ^a^	1.01	0.026	0.039	0.141	0.039
Mcal ME intake/kg gain	18.9 ^a^	17.5 ^a^	17.1 ^b^	18.5 ^a^	1.00	0.021	0.047	0.110	0.049

^a,b^ Means in the same row with different superscripts differ (*p* < 0.05). *p*-value is the observed significance level of the F-test for treatment; SEM = standard error of the mean. ^1^ The control diet based on (per kg DM): 600 g of concentrates feed mixture and 400 g berseem clover (*Trifolium alexandrinum*) (NSM0 diet). *Nigella sativa* seed meal was included at 7.8%, 11.7%, or 15.6% to replace soybean meal at 50% (NSM50 diet), 75% (NSM75 diet), or 100% (NSM100 diet), respectively. DCP = apparent digestible crude protein, DE = digestible energy, ME = metabolizable energy, SV = starch value, TDN = total digestible nutrients, DM = dry matter.

**Table 7:** Nutrient digestibility and diet nutritive value in Ossimi lambs fed diets with different levels of *Nigella sativa* seed meal replacing soybean meal as a protein source.

	Diet ^1^				SEM	*p*-Value			
	NSM0	NSM50	NSM75	NSM100		Diet	NSM0 vs. Others	Linear	Quadratic
Digestibility (g digested/g ingested)									
OM	0.735	0.746	0.754	0.710	0.0084	0.362	0.302	0.141	0.805
CP	0.768	0.762	0.768	0.726	0.0029	0.085	0.152	0.163	0.036
EE	0.745	0.776	0.820	0.735	0.0082	0.209	0.211	0.130	0.411
NFC	0.679	0.639	0.695	0.661	0.0094	0.126	0.235	0.955	0.749
NDF	0.751	0.756	0.766	0.726	0.0079	0.512	0.544	0.247	0.539
ADF	0.613	0.671	0.659	0.612	0.0069	0.388	0.195	0.202	0.354
Nutritive values (g/kg DM)									
Apparent SV	661	674	687	646	6.8	0.249	0.180	0.090	0.903
True SV	636	650	660	619	7.4	0.251	0.181	0.092	0.901
TDN	671	686	697	656	7.4	0.227	0.155	0.083	0.938
Apparent DCP	129	127	127	120	2.1	0.149	0.132	0.891	0.221
DE (Mcal/kg)	2.96	3.02	3.07	2.89	0.332	0.219	0.156	0.079	0.904
ME (Mcal/kg)	2.43	2.48	2.52	2.37	0.211	0.191	0.109	0.070	0.754

*p*-value is the observed significance level of the *F*-test for treatment; SEM = standard error of the mean. ^1^ The control diet based on (per kg DM): 600 g of concentrates feed mixture and 400 g berseem clover (*Trifolium alexandrinum*) (NSM0 diet). *Nigella sativa* seed meal was included at 7.8%, 11.7%, or 15.6% to replace soybean meal at 50% (NSM50 diet), 75% (NSM75 diet), or 100% (NSM100 diet), respectively. ADF = acid detergent fiber, CP = crude protein, apparent DCP = apparent digestible crude protein, DE = digestible energy, EE = ether extract, NDF = neutral detergent fiber, NFC = non-fibrous carbohydrates, ME = metabolizable energy, OM = organic matter, SV = starch value, TDN = total digestible nutrients.

**Table 8:** Blood measurements in Ossimi lambs fed diets with different levels of *Nigella sativa* seed meal replacing soybean meal as a protein source.

	Diet ^1^				SEM	*p*-Value			
	NSM0	NSM50	NSM75	NSM100		Diet	NSM0 vs. Others	Linear	Quadratic
Total protein (g/dL)	8.23	8.50	8.80	8.40	0.15	0.493	0.224	0.299	0.556
Albumin (g/dL)	4.58	4.68	4.75	4.68	0.071	0.224	0.270	0.095	0.368
Globulin (g/dL)	3.65	3.82	4.05	3.72	0.215	0.813	0.565	0.965	0.483
Urea-N (mg/dL)	34.7	34.3	35.0	37.0	2.37	0.366	0.204	0.618	0.326
Creatinine (mg/dL)	1.00	1.08	1.07	1.09	0.050	0.070	0.646	0.484	0.055
Glucose (mg/dL)	65.0	60.0	64.3	67.0	2.26	0.718	0.648	0.465	0.812
Alanine transaminase (IU/L)	15.0	15.3	15.8	15.3	0.79	0.090	0.060	0.031	0.820
Aspartate transaminase (IU/L)	93.3	95.0	91.7	94.0	3.76	0.361	0.282	0.785	0.145
Cholesterol (mg/dL)	98.0 ^a^	90.7 ^b^	89.3 ^b^	90.0 ^b^	1.18	0.036	0.039	0.028	0.884
Triglycerides (mg/dL)	49.0	47.3	45.7	44.3	2.91	0.374	0.170	0.300	0.430
High-density lipoprotein (mg/dL)	36.0 ^a^	30.3 ^b^	30.0 ^b^	31.0 ^b^	0.96	0.031	0.046	0.029	0.223
Antioxidant activity (%)	82.4 ^b^	91.3 ^a^	94.2 ^a^	97.2 ^a^	1.89	0.035	0.024	0.030	0.085

^a,b^ Means in the same row with different superscripts differ (*p* < 0.05). *p*-value is the observed significance level of the *F*-test for treatment; SEM = standard error of the mean. ^1^ The control diet based on (per kg DM): 600 g of concentrates feed mixture and 400 g berseem clover (*Trifolium alexandrinum*) (NSM0 diet). *Nigella sativa* seed meal was included at 7.8%, 11.7%, or 15.6% to replace soybean meal at 50% (NSM50 diet), 75% (NSM75 diet), or 100% (NSM100 diet), respectively.

**Table 9:** Economic evaluation of replacing soybean meal with different levels of *Nigella sativa* seed meal as a protein source in diets of Ossimi lambs.

	Diet ^1^				SEM	*p*-Value			
	NSM0	NSM50	NSM75	NSM100		Diet	NSM0 vs. Others	Linear	Quadratic
Total weight gain (kg/lamb/90 day)	19.0 ^b^	20.6 ^a^	21.7 ^a^	18.4 ^b^	0.48	0.001	0.034	<0.001	0.091
Feed intake (kg/lamb/90 day)	147.6	145.8	148.5	144.9	22.27	0.324	0.337	0.319	0.439
Cost of one kg DM of the diet (USD ^2^)	0.35 ^a^	0.31 ^b^	0.29 ^c^	0.28 ^d^	0.008	<0.001	<0.001	<0.001	<0.001
Cost of feed consumed (USD/lamb/90 day)	51.7 ^a^	45.2 ^b^	43.1 ^c^	40.6 ^d^	0.88	<0.001	<0.001	<0.001	<0.001
Total revenue (USD)	83.6 ^b^	90.6 ^a^	95.5 ^a^	81.0 ^b^	2.20	<0.001	<0.001	<0.001	<0.001
Net revenue (USD)	31.9 ^c^	45.4 ^b^	52.4 ^a^	40.4 ^b^	2.11	0.001	0.034	<0.001	0.091
Relative percentage of net revenue	100 ^c^	142.3 ^ab^	164.3 ^a^	126.6 ^bc^	2.39	<0.001	<0.001	<0.001	0.889

^a–d^ Means in the same row with different superscripts differ (*p* < 0.05). *p*-value is the observed significance level of the *F*-test for treatment; SEM = standard error of the mean. ^1^ The control diet based on (per kg DM): 600 g of concentrates feed mixture and 400 g berseem clover (*Trifolium alexandrinum*) (NSM0 diet). *Nigella sativa* seed meal was included at 7.8%, 11.7%, or 15.6% to replace soybean meal at 50% (NSM50 diet), 75% (NSM75 diet), or 100% (NSM100 diet), respectively. ^2^ All prices are in USD, equivalent to EGP 31 (31 Egyptian pounds) at the time of this study.

## Data Availability

The original contributions presented in the study are included in the article, further inquiries can be directed to the corresponding authors.
